# Virologic response to efavirenz-based first-line antiretroviral therapy in children with previous exposure to antiretrovirals to prevent mother-to-child transmission

**DOI:** 10.1371/journal.pone.0233693

**Published:** 2020-05-29

**Authors:** Patience Nyakato, Mary-Ann Davies, Karl-Gunter Technau, Geoffrey Fatti, Helena Rabie, Frank Tanser, Andrew Boulle, Robin Wood, Brian Eley, Shobna Sawry, Janet Giddy, Nosisa Sipambo, Louise Kuhn, Lee Fairlie, for the International epidemiology Database to Evaluate AIDS-Southern Africa (IeDEA-SA) Collaboration

**Affiliations:** 1 Center for Infectious Diseases Epidemiology and Research, School of Public Health an Family Medicine, Faculty of Health Sciences, University of Cape Town, Cape Town, Western Cape, South Africa; 2 Empilweni Services and Research Unit, Department of Paediatrics and Child Health, University of the Witwatersrand, Johannesburg, South Africa; 3 Kheth’Impilo AIDS Free Living, Cape Town, Western Cape, South Africa; 4 Division of Epidemiology and Biostatistics, Department of Global Health, Faculty of Medicine and Health Sciences, Stellenbosch University, Stellenbosch, Cape Town, Western Cape, South Africa; 5 University of Stellenbosch, Stellenbosch, Cape Town, Western Cape, South Africa; 6 Tygerberg Academic Hospital, Cape Town, Western Cape, South Africa; 7 Africa Health Research Institute, KwaZulu-Natal, Durban, South Africa; 8 Lincoln International Institute for Rural Health, University of Lincoln, Lincoln, England, United Kingdom; 9 School of Nursing and Public Health, University of KwaZulu-Natal, Durban, South Africa; 10 Centre for the AIDS Programme of Research in South Africa (CAPRISA), University of KwaZulu-Natal, Durban, South Africa; 11 Khayelitsha ART Program, Cape Town, Western Cape, South Africa; 12 Western Cape Department of Health, Cape Town, Western Cape, South Africa; 13 Gugulethu ART Program, Cape Town, Western Cape, South Africa; 14 Red Cross War Memorial Children’s Hospital, Cape Town, Western Cape, South Africa; 15 Department of Paediatrics and Child Health, University of Cape Town, Cape Town, Western Cape, South Africa; 16 Wits Reproductive Health and HIV Institute (Wits RHI), Faculty of Health Sciences, University of the Witwatersrand, Johannesburg, South Africa; 17 McCord Hospital, Durban, South Africa; 18 Department of Paediatrics and Child Health, Chris Hani Baragwanath Academic Hospital, University of Witwatersrand, Johannesburg, South Africa; 19 Gertrude H Sergievsky Center, College of Physicians and Surgeons and Department of Epidemiology, Mailman School of Public Health, Columbia University, New York, New York, United States of America; Consejo Superior de Investigaciones Cientificas, SPAIN

## Abstract

Efavirenz-based first-line regimens have been widely used for children ≥3 years of age starting antiretroviral therapy, despite possible resistance with prior exposure to non-nucleoside reverse transcriptase inhibitors for prevention of mother-to-child transmission (PMTCT). We used logistic regression to examine the association between PMTCT exposure and viral failure (VF) defined as two consecutive viral loads (VL)>1000 copies/ml between 6–18 months on ART. Children with previous nevirapine exposure for PMTCT were not at higher risk of VF compared to unexposed children (adjusted Odds Ratio (aOR): 0.79; 95% CI:0.56, 1.11).

## Introduction

While prevention of mother to child HIV transmission (PMTCT) has greatly reduced the number of new pediatric HIV infections, there are still 1.8 million children <15 years of age living with HIV both due to ongoing mother-to-child transmission, and survival of children living with HIV. Over 80% of these children live in sub Saharan Africa (SSA) [1]. Despite recommendations and guidelines by World Health Organisation (WHO) to treat all people living with HIV irrespective of age and CD4 count [2], optimal antiretroviral therapy (ART) dosing and formulations across all pediatric weight bands and age groups is still a challenge [3]. Metabolic and pharmacokinetic changes related to child development and puberty may require different dosing requirements for children compared to adults [4]. While dolutegravir (DTG) provides hope for simplification and harmonization of pediatric and adult regimens, it is still also not recommended for younger children below 30 kilograms (kgs) and for some children above three years of age [3–5].

In resource limited settings, efavirenz (EFV) will remain part of first-line pediatric regimens and is likely to continue to be relatively widely used [6]. In addition, EFV may be preferred in children requiring rifampicin-based tuberculosis co-treatment [7] and among female adolescents of child bearing age who are not on any, or have inconsistent use of contraception [8]. EFV has been recommended for older children (>3 years) and adults for several years due to its advantages for long term maintenance, once daily dosing, simplification of co-treatment for tuberculosis and preserving alternative drugs for second-line [9].

However, the number of children living with HIV initiating first-line non-nucleoside reverse transcriptase inhibitor (NNRTI) treatment with pre-treatment drug resistance (PDR) due to prior exposure to maternal or infant PMTCT regimens is a significant challenge [9, 10]. Both NVP and EFV have a low genetic barrier to resistance and nevirapine (NVP) remains part of WHO-recommended infant prophylaxis during breastfeeding. Y181C is the most commonly selected mutation following NVP exposure which confers high-level resistance to NVP and low-level resistance to EFV [10]. Kityo *et al*. reported that 1 in 6 children initiating first line ART (164/278 on EFV, 104/278 on NVP and 10/278 on protease inhibitor (PI)) in Uganda had PDR; children with PDR were 15 times more likely to experience virologic failure compared to those without. Children with prior or unknown PMTCT exposure were more likely to have PDR, and although PDR proportions were high, they may have been underestimated due to archived resistance in older children [11]. Among Nigerian children initiating treatment, 16% had PDR and among these 33% experienced treatment failure by 24 months on treatment [12].

The Nevirapine Resistance trial (NEVEREST III) showed non-inferior virologic outcomes (viral failure or rebound) for children < 3years old initiating lopinavir-based first-line and switching to an EFV-based regimen at 3–5 years of age [9]. In both of these studies very few children had extended infant NVP prophylaxis, so the prevalence of PDR and subsequent virologic outcomes (virologic suppression or failure) on EFV-based first-line in this context are not known.

Our study aimed to investigate the association between PMTCT exposure and viral failure in children aged at least three years starting EFV-based ART using routine data from the International epidemiology Database to Evaluate AIDS-Southern Africa (IeDEA-SA) collaboration.

## Materials and methods

### Study setting and population

We used data from IeDEA-SA, a regional collaboration of adult and pediatric HIV treatment programs [13]. We included all children living with HIV aged 3–13 years who initiated EFV-based ART between 2004 and 2014 at 10 public sector ART programs in South Africa and who had at least two viral load (VL) measurements from six-18 months on ART.

### Exposure and outcome definition

PMTCT exposure status was recorded as exposed, unexposed or unknown. Actual PMTCT regimens were not consistently recorded but the data contained a variable differentiating between those that had been exposed to PMTCT or not based on mother-to child data linkage. Maternal and child PMTCT regimens from relevant South African national and provincial guidelines over the study period are shown in S1a Table and S1b Table in S1 File.

We defined viral failure (VF) as having two consecutive VLs ≥ 1000 copies/ml in the 6–18 months after ART initiation. We further present results for the outcome of VF defined as two consecutive VLS ≥ 400 copies/ml in the follow up period. We also present results on virologic non-suppression in S5 Table and S6 Table in S1 File for both cut offs respectively. This was defined as the maximum viral load above 1000 copies/ml in the 6–18 months window to occur (one line per patient was considered). For children with more than 2 values, the worst (highest) value was considered as either suppressed or unsuppressed depending on whether or not it was above 1000 copies/ml.

### Statistical analysis

Descriptive statistics (means (standard deviation), medians (interquartile range) and proportions) were used to summarize patient characteristics. We examined the association between PMTCT exposure and VF between 6–18 months on ART using logistic regression and adjusting for other patient characteristics including calendar year of ART start. Since no PMTCT was available in the public sector in South Africa before 2000, we assumed that children with unknown PMTCT exposure born before 2000 were PMTCT unexposed.

In sensitivity analyses for those born during or after 2000, we assumed PMTCT exposure for those with unknown exposure was either: (1) all exposed as per South African PMTCT guidelines at the time of the child’s birth or (2) all unexposed. Results presented are based on complete case analysis(CCA). Multiple imputation (MI) of missing data on WHO stage and immunosuppression using 10 imputed datasets was also done and the results were combined using Rubin’s rules. There was no difference in the results between the CCA and MI for both outcomes of VF (2 consecutive VL ≥1000 copies/ml or 400 copies/ml respectively) (S2 Table–S4 Table in S1 File). All analysis was done using STATA version 15.1.

### Ethics

IeDEA-SA cohorts have obtained ethical approval to collect and transfer anonymized data through their respective Institutional Review Boards (IRBs). The IeDEA-SA data centre has approval from the University of Cape Town’s IRB (Human Research Ethics Committee(HREC)) to receive and analyse these anonymised data.

## Results

Of the 7,896 children included in the analysis, 3,948 (50%) were girls with a median age at ART start of 7.5 (IQR: 5.3, 10.2) years. Over two thirds 5,282/7,896 (66.9%) of the children had initiated ART with World Health Organisation (WHO) clinical stage 3 or 4 disease, and 2,320 (40.1%) had WHO-defined severe immunosuppression (Table 1).

**Table 1:** Characteristics of children stratified by three prevention of mother-to-child transmission (PMTCT) exposure scenarios:- i) according to clinic records, “unknown” category allocated to ii) PMTCT according to national guidelines at the year of birth (YoB) and iii) not receiving PMTCT.

	PMTCT exposure status according to the clinic records^$^			Unknown PMTCT allocated to PMTCT exposure according to the year of birth^$^			Unknown PMTCT allocated to no PMTCT exposure^$^		
	No (N = 5909)	Yes (N = 529)	Unknown (N = 1458)	No (N = 6483)	Yes (N = 1413)	Total (N = 7896)	No (N = 7372)	Yes (N = 524)	Total (N = 7896)
Sex, n (%)									
Female	3093 (50.6)	261 (49.3)	1007 (49.5)	3283 (50.6)	682 (48.3)	3965 (50.2)	3706 (50.3)	259 (49.5)	3965 (50.2)
WHO Stage^a^, n (%)									
Stages 1&2	1372 (36.1)	34 (15.0)	274 (26.4)	1472 (35.2)	208 (23.4)	1680 (33.1)	1650 (34.0)	30 (13.6)	1680 (33.1)
Stages 3&4	2434 (64.0)	192 (85.0)	766 (73.7)	2711 (64.8)	681 (76.6)	3392 (66.9)	3200 (66.0)	192 (86.5)	3392 (66.9)
WHO defined immunosuppression*, n (%)									
No	2393 (57.4)	296 (68.4)	773 (65.3)	2682 (57.7)	780 (68.6)	3462 (59.9)	3169 (59.2)	293 (68.1)	3462 (59.9)
Yes	1773 (42.6)	137 (31.6)	410 (34.6)	1963 (42.3)	357 (31.4)	2320 (40.1)	2183 (40.8)	137 (31.9)	2320 (40.1)
Year of antiretroviral therapy (ART) start, n (%)									
2004–2005	925 (15.1)	20 (3.8)	319 (15.7)	979 (15.1)	75 (5.3)	1054 (13.4)	1034 (14.0)	20 (3.8)	1054 (13.4)
2006–2007	1076 (17.6)	81 (15.3)	530 (26.0)	1176 (18.1)	252 (17.9)	1428 (18.1)	1348 (18.3)	80 (15.3)	1428 (18.1)
2008–2009	1236 (20.2)	159 (30.1)	563 (27.7)	1338 (20.6)	417 (29.5)	1755 (22.2)	1598 (21.7)	157 (30.0)	1755 (22.2)
2010–2011	1252 (20.5)	155 (29.3)	395 (19.4)	1328 (20.5)	386 (27.3)	1714 (21.7)	1560 (21.2)	154 (29.5)	1714 (21.7)
2012–2014	1625 (26.6)	114 (21.6)	228 (11.2)	1663 (25.7)	282 (20.0)	1945 (24.6)	1833 (24.9)	112 (21.4)	1945 (24.6)
Year of birth, n (%)									
Before 2000	1668 (31.3)	0 (0.0)	782 (38.4)	2450 (37.8)	0 (0.0)	2450 (1.0)	2450 (33.2)	0 (0.0)	2450 (31.0)
2000–2003	2184 (40.9)	239 (45.2)	829 (40.7)	2554 (39.4)	698 (49.4)	3252 (41.2)	3016 (40.9)	236 (45.1)	3252 (41.2)
2004–2005	685 (12.9)	171 (32.3)	271 (13.3)	685 (10.6)	442 (31.2)	1127 (14.3)	958 (13.0)	169 (32.3)	1127 (14.3)
2006–2007	479 (9.0)	69 (13.0)	111 (5.5)	479 (7.4)	180 (12.8)	659 (8.4)	591 (8.0)	68 (13.0)	659 (8.4)
2008–2009	248 (4.7)	46 (8.7)	35 (1.7)	248 (3.8)	81 (5.7)	329 (4.2)	283 (3.8)	46 (8.8)	329 (4.2)
2010 and beyond	68 (1.3)	4 (0.8)	7 (0.3)	68 (1.1)	11 (0.8)	79 (1.0)	75 (1.0)	4 (0.8)	79 (1.0)
Weight for age Z- score, (Mean (SD))	-1.68 (1.30)	-1.61 (1.22)	-1.39 (1.22)	-1.48 (1.30)	-1.28 (1.26)	-1.43 (1.29)	-1.44 (1.31)	-1.37 (1.21)	-1.43 (1.29)
Age at ART start, (years, median (IQR))	8.29 (5.89, 10.71)	5.45 (3.98, 6.63)	5.78 (4.29, 7.69)	8.24 (5.89, 10.65)	5.16 (3.91, 6.73)	7.52 (5.25, 10.18)	7.81 (5.42, 10.33)	5.08 (4.0, 6.63)	7.52 (5.25, 10.18)
PMTCT infant drug, n (%)									
sdNVP		480 (90.6)			1329 (94.1)			479 (91.6)	
sdNVP+AZT		42 (7.9)			76 (5.4)			42 (8.0)	
NVP 6 weeks		2 (0.4)			7 (0.5)			2 (0.4)	
Unknown		5 (1.0)	2035 (100.0)						
Maternal PMTCT regimen, n (%)									
sdNVP	0 (0.0)	474 (89.6)	0 (0.0)		1285 (91.0)			474 (90.6)	
sdNVP + AZT	0 (0.0)	45 (8.5)	0 (0.0)		126 (8.9)			45 (8.6)	
AZT only	0 (0.0)	10 (1.9)	2035 (100.0)	1 (0.0)	0 (0.0)		1 (0.01)	0 (0.0)	

^$^Children born before 2000 with unknown PMTCT exposure (n = 782) assumed to get no PMTCT as no PMTCT was available in the public sector before 2000.

^a^Missing observations for 2824/ 7896 (35.8%),

*Missing observations for 2114/7896 (26.8%) of children of children

Recorded PMTCT exposure was: 5,909 (74.8%) unexposed, 529 (6.7%) exposed and 1,458 (18.5%) unknown. After assuming that those with unknown exposure received PMTCT according to the year of birth, there were a total of 17.9% (1,413/7896) exposed. Overall, VF was experienced by 1,224/7896 (15.5%) in the period of 6–18 months on ART, and among these, 1,021 (83.4%) had no PMTCT exposure, 61 (5.0%) had been exposed to PMTCT and 142 (11.6%) had unknown exposure to PMTCT.

After adjusting for immunosuppression, calendar year at ART start, age at ART start as a continuous variable and the year of birth (Table 2), children with previous PMTCT exposure did not have higher odds of experiencing VF compared to unexposed children (adjusted Odds Ratio (aOR): 0.79; 95% CI: 0.56, 1.11). In sensitivity analyses, after assuming children with unknown PMTCT exposure received PMTCT according to South African guidelines at the time and adjusting for the above covariates, there continued to be no evidence of increased odds of VF in PMTCT exposed compared to unexposed children (aOR:0.66; 95%CI: 0.52,0.85). Likewise, if those of unknown PMTCT exposure were assumed to have received no PMTCT, there was also no evidence of increased odds of VF (aOR:0.91; 95% CI: 0.65, 1.27) respectively. Furthermore, sensitivity analysis looking at a cut off of ≥ 400 copies/ml for VF yielded similar results (S3 Table in S1 File).

**Table 2:** Univariable and multivariable models of association between prevention of mother-to-child transmission (PMTCT) exposure and viral failure (VF) defined as two consecutive viral load (VL) ≥1000 copies/ml between 6–18 months on ART: Analysis based on 5782 patients.

Patient characteristics at ART start	Univariable associations		No assumptions made for unknown PMTCT^$^		Assumption I: Unknown got PMTCT according to year of birth		Assumption II: Unknown assumed not to have got any PMTCT	
	Crude OR*	95% CI**	Adjusted OR*	95% CI**	Adjusted OR*	95% CI**	Adjusted OR*	95% CI**
PMTCT exposure status								
No	1		1		1		1	
Yes	0.62	0.47, 0.82	0.79	0.56, 1.11	0.66	0.52, 0.85	0.91	0.65, 1.27
Unknown	0.52	0.43, 0.62	0.55	0.43, 0.69				
Sex								
Male	1		1		1		1	
Female	0.96	0.85, 1.08	0.94	0.81, 1.09	0.94	0.81, 1.09	0.94	0.81, 1.10
WHO Stage								
Stages 1&2	1							
Stages 3&4	1.06	0.91, 1.24						
WHO-defined Immunosuppression								
No	1		1		1		1	
Yes	2.14	1.82, 2.53	1.75	1.50, 2.04	1.75	1.51, 2.04	1.76	1.51, 2.04
Calendar Year of ART start								
2004–2005	1		1		1		1	
2006–2007	0.87	0.69, 1.09	0.88	0.68, 1.15	0.86	0.66, 1.12	0.83	0.64, 1.08
2008–2009	0.64	0.51, 0.81	0.62	0.45, 0.84	0.60	0.44, 0.82	0.57	0.42, 0.77
2010–2011	1.45	1.18, 1.79	1.54	1.08, 2.17	1.48	1.05, 2.09	1.39	0.99, 1.96
2012–2014	1.34	1.09, 1.64	1.28	0.82, 1.97	1.24	0.80, 1.91	1.19	0.77, 1.84
Year of birth								
Before 2000	1		1		1		1	
2000–2003	0.82	0.71, 0.94	0.84	0.64, 1.10	0.83	0.64, 1.09	0.80	0.61, 1.05
2004–2005	0.60	0.49, 0.74	0.72	0.46, 1.13	0.78	0.50, 1.21	0.73	0.47, 1.13
2006–2007	0.72	0.57, 0.92	0.85	0.49, 1.46	0.92	0.53, 1.58	0.89	0.52, 1.53
2008 and beyond	0.76	0.57, 1.02	1.00	0.50, 1.99	1.12	0.56, 2.21	1.11	0.56 2.19
Age at ART start in years	1.12	1.10, 1.15	1.05	0.99 1.11	1.06	1.00, 1.11	1.07	1.02, 1.13

^$^Children born before 2000 with unknown PMTCT exposure (n = 782) assumed to get no PMTCT as no PMTCT available in the public sector before 2000

*Odds Ratios.

** Confidence Intervals.

^#^All models have been adjusted for sex, immunosuppression at ART start, calendar of ART start, year of birth and age at ART start

## Discussion

Our study showed no evidence of an increased risk of VF among children who were exposed to PMTCT starting EFV-based ART at ≥3 years of age. There continued to be no evidence of an increased risk in a sensitivity analysis assuming children with unknown PMTCT exposure either received no PMTCT or received the PMTCT regimen available at the time of their birth.

Our results concur with the NEVEREST III randomized clinical trial (RCT) which randomized PMTCT-exposed children to an EFV-based regimen or to continue lopinavir/ritonavir-based ART [9]. This trial showed no increased risk of VF when virologically-suppressed children above three years old on lopinavir-based regimens with prior single dose NVP-based PMTCT exposure were switched to EFV-based regimens. Podjanee *et al* in Thailand also found a similar association of no difference in VF risk based on PMTCT exposure, with a quarter of the children with and without PMTCT exposure experiencing VF during the study period [14].

As children had to be at least three years old at ART start to initiate an EFV-based regimen according to South African guidelines, there were only 11 children with documented PMTCT exposure after the introduction of extended infant NVP prophylaxis, hence we were unable to determine the effect of prolonged infant NVP on outcomes for subsequent EFV-based ART, although none of the 11 children experienced VF. Nonetheless, it is hypothesized that since a single mutation confers EFV resistance and almost all single-dose NVP-exposed infants harbour one of these mutations, longer durations of infant NVP are unlikely to worsen subsequent EFV-based ART outcomes. While some studies have suggested worse outcomes for PMTCT-exposed children on NNRTI-based ART, most PMTCT-exposed children in these studies would have been treated with NVP-based rather than EFV-based ART [15, 16]. Regardless of our findings, the high prevalence of HIV drug resistance to NNRTIs, as a result of the low genetic barrier to resistance, is a major challenge with NNRTI use, specifically EFV, and children receiving EFV should be closely monitored for adherence and viral suppression.

Even with the increasing rollout of DTG for treatment of children living with HIV, many resource-limited settings in SSA and Asia will continue to use EFV in younger children and women or adolescent girls of child bearing age. In addition to the concerns regarding resistance, EFV is reported to have adverse side effects like central nervous system toxicity although this is more mild in children than it is in adults [17]. There are also additional potential side effects such as impaired concentration, skin rash, dizziness, sleep disturbances and anxiety [18, 19]. PI-based first line therapy may offer better outcomes compared to the NNRTIs [20–22]. It is important to have options for first line ART in children and challenges with lopinavir/ritonavir include poor palatability, increased abdominal side effects (diarrhoea and nausea), drug-drug interactions with rifampicin co-treatment and the increased cost and lack of improved paediatric formulations.

Our study had several limitations. Although we adjusted for pre-ART patient characteristics and calendar time, there may be other factors (structural, clinical or psychosocial) related to access to PMTCT that are also associated with better ART outcomes. While this may partially explain the reduced VF associated with PMTCT exposure when assuming that all children with unknown PMTCT exposure received PMTCT according to the year of birth, these factors may also have introduced confounding and potentially masked an association between PMTCT and worse ART response. In addition, since we relied on routinely collected cohort data, we did not have data on other variables that may impact the outcome such as adherence, breastfeeding and cotrimoxazole use, so could not adjust for these variables. We also do not have data regarding the reasons for failing PMTCT. We also only included children that had ≥2 VL measurement between 6 and 18 months on ART. This may have introduced selection bias given there may be children who potentially experienced VF but had <2 VL results.

While the use of data from routine care settings in South Africa makes our results generalizable, the recording of PMTCT exposure in routine program data was incomplete, although data completeness improved in more recent calendar years. Additionally, actual maternal and infant regimens were not recorded except for two facilities where we could link the child to the maternal ART file. Notwithstanding, analyses using recorded PMTCT exposure and assumed exposure based on year of birth both did not show an adverse effect of PMTCT exposure on VF. Further, the resistance profile of children in a Ugandan study suggests that a large proportion of children with unknown PMTCT exposure were likely exposed, supporting our sensitivity analysis approach [11].

In conclusion, our finding of no evidence of increased risk of VF among PMTCT exposed children initiating first line EFV-based regimens is reassuring given that EFV-based regimens have been widely used in children living with HIV, and may continue to be used until DTG is registered and accessible for younger children, and in children requiring tuberculosis co-treatment. However, the impact of extended infant NVP prophylaxis on the virologic efficacy of subsequent EFV-based ART remains to be fully assessed.

## Supporting information

S1 File Supplementary files for the association between virologic response and prevention of mother to child transmission (PMTCT) exposure among children initiated on efavirenz (EFV) based regimen. (DOCX)
