# Supplementary material for: Virologic response to efavirenz-based first-line antiretroviral therapy in children with previous exposure to antiretrovirals to prevent mother-to-child transmission
Source: PLoS One. 2020 May 29;15(5):e0233693. doi: 10.1371/journal.pone.0233693 (PMC7259572; doi:10.1371/journal.pone.0233693)
Supplement: S1 File — (DOCX) [file pone.0233693.s001.docx]

**Supplementary files for the association between virologic response and prevention of mother to child transmission (PMTCT) exposure among children initiated on efavirenz (EFV) based regimen.**

**S1a* Table: Maternal prevention of mother to child transmission (PMTCT) guidelines in South Africa over the study period**

| Period | Regimen |
| --- | --- |
| <January 1999 | No PMTCT |
| Feb 2002-Feb 2008 | Single dose nevirapine (sdNVP) |
| 2004 onwards | CD4 and clinically-based antiretroviral therapy (ART) indications |
| Feb 2008 | Zidovudine (AZT) from 28 weeks; sdNVP |
| April 2010 | AZT from 14 weeks. Truvada and sdNVP |
| April 2013 onwards | Efavirenz-emtricitabine-tenofovir |

**S1b* Table: Infant prevention of mother-to-child transmission guidelines over the study period**

| Period | Regimen |
| --- | --- |
| < January 1999 | No PMTCT |
| Feb 2002-Feb 2008 | sdNVP |
| Feb 2008-March 2010 | sdNVP + (AZT) |
| April 2010 onwards | Daily nevirapine (NVP) at least 6 weeks |

*These tables reflect the National Department of Health guidelines over the study period. Differences in guidelines implemented in the Western Cape are accounted for in the assumptions.

**S2 Table: Univariable and multivariable models of association between prevention of mother-to-child transmission exposure and VF defined as two consecutive VL >1000 copies/ml between 6-18 months on ART: Multiple imputation results**

|  | **Univariable associations** | | **No assumptions made for unknown PMTCT^$^** | | **Assumption I: Unknown got PMTCT according to yob** | | **Assumption II: Unknown assumed not to have got any PMTCT** | |
| --- | --- | --- | --- | --- | --- | --- | --- | --- |
| **Patient characteristics at ART start** | **Crude OR^*^** | **95% CI^**^** | **Adjusted OR^**^** | **95% CI^***^** | **Adjusted OR^**^** | **95% CI^***^** | **Adjusted OR^**^** | **95% CI^***^** |
| **PMTCT exposure status** |  |  |  |  |  |  |  |  |
| No | 1 |  | 1 |  | 1 |  | 1 |  |
| Yes | 0.62 | 0.47, 0.82 | 0.85 | 0.63, 1.13 | 0.76 | 0.62, 0.92 | 0.95 | 0.71, 1.26 |
| Unknown | 0.52 | 0.43, 0.62 | 0.62 | 0.51, 0.75 |  |  |  |  |
| **Sex** |  |  |  |  |  |  |  |  |
| Male | 1 |  | 1 |  | 1 |  | 1 |  |
| Female | 0.96 | 0.85, 1.08 | 0.95 | 0.84, 1.07 | 0.95 | 0.84, 1.07 | 0.95 | 0.83, 1.08 |
| **WHO Stage** |  |  |  |  |  |  |  |  |
| Stages 1&2 | 1 |  |  |  |  |  |  |  |
| Stages 3&4 | 1.06 | 0.91, 1.24 |  |  |  |  |  |  |
| **WHO-defined Immunosuppression** |  |  |  |  |  |  |  |  |
| No | 1 |  | 1 |  | 1 |  | 1 |  |
| Yes | 2.14 | 1.82, 2.53 | 2.06 | 1.72, 2.47 | 2.05 | 1.72, 2.46 | 2.05 | 1.71, 2.45 |
| **Calendar Year of ART start** |  |  |  |  |  |  |  |  |
| 2004-2005 | 1 |  | 1 |  | 1 |  | 1 |  |
| 2006-2007 | 0.87 | 0.69, 1.09 | 0.88 | 0.69, 1.13 | 0.87 | 0.68, 1.11 | 0.85 | 0.67, 1.08 |
| 2008-2009 | 0.64 | 0.51, 0.81 | 0.66 | 0.51, 0.87 | 0.65 | 0.50, 0.86 | 0.63 | 0.48, 0.83 |
| 2010-2011 | 1.45 | 1.18, 1.79 | 1.60 | 1.18, 2.17 | 1.56 | 1.15, 2.12 | 1.51 | 1.11, 2.04 |
| 2012-2014 | 1.34 | 1.09, 1.64 | 1.43 | 0.97, 2.09 | 1.41 | 0.96, 2.06 | 1.37 | 0.94, 2.00 |
| **Year of birth** |  |  |  |  |  |  |  |  |
| Before 2000 | 1 |  | 1 |  | 1 |  | 1 |  |
| 2000-2003 | 0.82 | 0.71, 0.94 | 0.91 | 0.72, 1.14 | 0.89 | 0.71, 1.12 | 0.87 | 0.70, 1.10 |
| 2004-2005 | 0.60 | 0.49, 0.74 | 0.71 | 0.49, 1.04 | 0.74 | 0.51, 1.08 | 0.71 | 0.49, 1.03 |
| 2006-2007 | 0.72 | 0.57, 0.92 | 0.79 | 0.50, 1.25 | 0.83 | 0.52, 1.31 | 0.82 | 0.52, 1.30 |
| 2008 and beyond | 0.76 | 0.57, 1.02 | 0.84 | 0.48, 1.48 | 0.89 | 0.51, 1.57 | 0.90 | 0.51 1.58 |
| **Age at ART start in years** | 1.12 | 1.10, 1.15 | 1.07 | 1.02 1.12 | 1.07 | 1.02, 1.12 | 1.08 | 1.04, 1.13 |

^$^Children born before 2000 with unknown PMTCT exposure (n=782) assumed to get no PMTCT as no PMTCT available in the public sector before 2000. *Crude odds ratio ** Adjusted odds ratio, *** 95% Confidence intervals

**S3 Table: Univariable and multivariable models of association between prevention of mother-to-child transmission exposure and VF defined as two consecutive VL >400 copies/ml between 6-18 months on ART: N=5782**

|  | **Univariable associations** | | **No assumptions made for unknown PMTCT^$^** | | **Assumption I: Unknown got PMTCT according to yob** | | **Assumption II: Unknown assumed not to have got any PMTCT** | |
| --- | --- | --- | --- | --- | --- | --- | --- | --- |
| **Patient characteristics at ART start** | **Crude OR^*^** | **95% CI^**^** | **Adjusted OR^**^** | **95% CI^***^** | **Adjusted OR^**^** | **95% CI^***^** | **Adjusted OR^**^** | **95% CI^***^** |
| **PMTCT exposure status** |  |  |  |  |  |  |  |  |
| No | 1 |  | 1 |  | 1 |  | 1 |  |
| Yes | 0.70 | 0.55, 0.90 | 0.84 | 0.63, 1.12 | 0.69 | 0.56, 0.85 | 0.95 | 0.71, 1.27 |
| Unknown | 0.56 | 0.47, 0.66 | 0.59 | 0.48, 0.72 |  |  |  |  |
| **Sex** |  |  |  |  |  |  |  |  |
| Male | 1 |  | 1 |  | 1 |  | 1 |  |
| Female | 0.96 | 0.86, 1.08 | 0.93 | 0.82, 1.07 | 0.95 | 0.82, 1.07 | 0.94 | 0.82, 1.07 |
| **WHO Stage** |  |  |  |  |  |  |  |  |
| Stages 1&2 | 1 |  |  |  |  |  |  |  |
| Stages 3&4 | 1.04 | 0.90, 1.20 |  |  |  |  |  |  |
| **WHO-defined Immunosuppression** |  |  |  |  |  |  |  |  |
| No | 1 |  | 1 |  | 1 |  | 1 |  |
| Yes | 1.89 | 1.61, 2.21 | 1.87 | 1.59, 2.19 | 1.86 | 1.58, 2.18 | 1.85 | 1.58, 2.18 |
| **Calendar Year of ART start** |  |  |  |  |  |  |  |  |
| 2004-2005 | 1 |  | 1 |  | 1 |  | 1 |  |
| 2006-2007 | 0.93 | 0.75, 1.15 | 0.93 | 0.73, 1.19 | 0.92 | 0.72, 1.17 | 0.89 | 0.70, 1.13 |
| 2008-2009 | 0.68 | 0.55, 0.85 | 0.68 | 0.52, 0.90 | 0.67 | 0.51, 0.89 | 0.64 | 0.49, 0.84 |
| 2010-2011 | 1.51 | 1.24, 1.84 | 1.55 | 1.13, 2.13 | 1.53 | 1.11, 2.09 | 1.43 | 1.05, 1.97 |
| 2012-2014 | 1.33 | 1.10, 1.62 | 1.23 | 0.82, 1.83 | 1.21 | 0.82, 1.81 | 1.17 | 0.79, 1.74 |
| **Year of birth** |  |  |  |  |  |  |  |  |
| Before 2000 | 1 |  | 1 |  | 1 |  | 1 |  |
| 2000-2003 | 0.85 | 0.74, 0.97 | 0.90 | 0.70, 1.15 | 0.89 | 0.70, 1.13 | 0.86 | 0.67, 1.10 |
| 2004-2005 | 0.69 | 0.57, 0.83 | 0.84 | 0.57, 1.25 | 0.90 | 0.61, 1.33 | 0.85 | 0.57, 1.25 |
| 2006-2007 | 0.82 | 0.65, 1.02 | 0.94 | 0.57, 1.53 | 1.00 | 0.61, 1.62 | 0.98 | 0.61, 1.60 |
| 2008 and beyond | 0.85 | 0.65, 1.11 | 1.09 | 0.60, 2.00 | 1.19 | 0.65, 2.17 | 1.20 | 0.66 2.19 |
| **Age at ART start in years** | 1.10 | 1.08, 1.12 | 1.05 | 1.00 1.10 | 1.05 | 1.00, 1.10 | 1.06 | 1.01, 1.12 |

^$^Children born before 2000 with unknown PMTCT exposure (n=782) assumed to get no PMTCT as no PMTCT available in the public sector before 2000. *Crude odds ratio ** Adjusted odds ratio, *** 95% Confidence intervals

**S4 Table: Univariable and multivariable models of association between prevention of mother-to-child transmission exposure and VF defined as two consecutive VL >400 copies/ml between 6-18 months on ART: Multiple imputation results**

|  | **Univariable associations** | | **No assumptions made for unknown PMTCT^$^** | | **Assumption I: Unknown got PMTCT according to yob** | | **Assumption II: Unknown assumed not to have got any PMTCT** | |
| --- | --- | --- | --- | --- | --- | --- | --- | --- |
| **Patient characteristics at ART start** | **Crude OR*** | **95% CI^***^** | **Adjusted OR^**^** | **95% CI^***^** | **Adjusted OR^**^** | **95% CI^***^** | **Adjusted OR^**^** | **95% CI^***^** |
| **PMTCT exposure status** |  |  |  |  |  |  |  |  |
| No | 1 |  | 1 |  | 1 |  | 1 |  |
| Yes | 0.70 | 0.55, 0.90 | 0.89 | 0.68, 1.15 | 0.77 | 0.64, 0.93 | 0.97 | 0.76, 1.28 |
| Unknown | 0.56 | 0.47, 0.66 | 0.65 | 0.54, 0.77 |  |  |  |  |
| **Sex** |  |  |  |  |  |  |  |  |
| Male | 1 |  | 1 |  | 1 |  | 1 |  |
| Female | 0.96 | 0.86, 1.08 | 0.95 | 0.85, 1.07 | 0.95 | 0.85, 1.07 | 0.95 | 0.85, 1.07 |
| **WHO Stage** |  |  |  |  |  |  |  |  |
| Stages 1&2 | 1 |  |  |  |  |  |  |  |
| Stages 3&4 | 1.04 | 0.90, 1.20 |  |  |  |  |  |  |
| **WHO-defined Immunosuppression** |  |  |  |  |  |  |  |  |
| No | 1 |  | 1 |  | 1 |  | 1 |  |
| Yes | 1.89 | 1.61, 2.21 | 1.86 | 1.58, 2.19 | 1.85 | 1.57, 2.19 | 1.85 | 1.58, 2.18 |
| **Calendar Year of ART start** |  |  |  |  |  |  |  |  |
| 2004-2005 | 1 |  | 1 |  | 1 |  | 1 |  |
| 2006-2007 | 0.93 | 0.75, 1.15 | 0.95 | 0.76, 1.19 | 0.93 | 0.75, 1.18 | 0.92 | 0.73, 1.15 |
| 2008-2009 | 0.68 | 0.55, 0.85 | 0.71 | 0.55, 0.91 | 0.70 | 0.54, 0.90 | 0.67 | 0.52, 0.87 |
| 2010-2011 | 1.51 | 1.24, 1.84 | 1.63 | 1.22, 2.17 | 1.61 | 1.21, 2.14 | 1.55 | 1.16, 2.06 |
| 2012-2014 | 1.33 | 1.10, 1.62 | 1.38 | 0.97, 1.98 | 1.37 | 0.96, 1.96 | 1.34 | 0.93, 1.91 |
| **Year of birth** |  |  |  |  |  |  |  |  |
| Before 2000 | 1 |  | 1 |  | 1 |  | 1 |  |
| 2000-2003 | 0.85 | 0.74, 0.97 | 0.92 | 0.74, 1.15 | 0.91 | 0.74, 1.13 | 0.89 | 0.72, 1.11 |
| 2004-2005 | 0.69 | 0.57, 0.83 | 0.78 | 0.55, 1.10 | 0.81 | 0.57, 1.14 | 0.78 | 0.55, 1.10 |
| 2006-2007 | 0.82 | 0.65, 1.02 | 0.85 | 0.55, 1.31 | 0.88 | 0.57, 1.36 | 0.88 | 0.57, 1.35 |
| 2008 and beyond | 0.85 | 0.65, 1.11 | 0.90 | 0.53, 1.52 | 0.95 | 0.56, 1.60 | 0.95 | 0.56 1.61 |
| **Age at ART start in years** | 1.10 | 1.08, 1.12 | 1.05 | 1.01 1.10 | 1.06 | 1.01, 1.10 | 1.06 | 1.02, 1.11 |

^$^Children born before 2000 with unknown PMTCT exposure (n=782) assumed to get no PMTCT as no PMTCT available in the public sector before 2000. *Crude odds ratio ** Adjusted odds ratio, *** 95% Confidence intervals

**NB:** In total 1460/7896 (18.5%) experienced viral failure (Two consecutive viral loads above 400 copies/ml). Among these 1,198/7896 were not exposed to PMTCT, 80 had been exposed and 182 had unknown PMTCT exposure.

**S5 Table: Univariable and multivariable models of association between PMTCT exposure and VL >1000 copies/ml (viral non-suppression) between 6-18 months on ART: N= 5872 patients.**

|  | **Univariable models** | | **No assumptions made for unknown PMTCT^$^** | | **Assumption I: Unknown got according to date of birth** | | **Assumption II: Unknown did not get any PMTCT** | |
| --- | --- | --- | --- | --- | --- | --- | --- | --- |
| **Patient characteristics at ART start** | **Crude OR** | **95% CI^**^** | **Adjusted OR^*^** | **95% CI^**^** | **Adjusted OR^*^** | **95% CI^**^** | **Adjusted OR^*^** | **95% CI^**^** |
| **PMTCT exposure status** |  |  |  |  |  |  |  |  |
| No | 1 |  | 1 |  | 1 |  | 1 |  |
| Yes | 1.00 | 0.81, 1.24 | 1.32 | 0.89, 1.94 | 0.97 | 0.76, 1.24 | 1.36 | 0.92, 2.00 |
| Unknown | 0.75 | 0.65, 0.85 | 0.84 | 0.67, 1.07 |  |  |  |  |
| **Sex** |  |  |  |  |  |  |  |  |
| Male | 1 |  | 1 |  | 1 |  | 1 |  |
| Female | 0.86 | 0.78, 0.96 | 0.86 | 0.72, 1.02 | 0.86 | 0.72, 1.03 | 0.86 | 0.72, 1.03 |
| **WHO Stage** |  |  |  |  |  |  |  |  |
| Stages 1&2 | 1 |  |  |  |  |  |  |  |
| Stages 3&4 | 1.10 | 0.06, 1.27 |  |  |  |  |  |  |
| **WHO-defined Immunosuppression** |  |  |  |  |  |  |  |  |
| No | 1 |  | 1 |  | 1 |  | 1 |  |
| Yes | 3.07 | 2.66, 3.55 | 3.70 | 3.02, 4.52 | 3.69 | 3.02, 4.52 | 3.72 | 3.04, 4.55 |
| **Calendar Year of ART start** |  |  |  |  |  |  |  |  |
| 2004-2005 | 1 |  | 1 |  | 1 |  | 1 |  |
| 2006-2007 | 0.94 | 0.76, 1.16 | 0.86 | 0.56, 1.30 | 0.86 | 0.56, 1.30 | 0.84 | 0.55, 1.27 |
| 2008-2009 | 1.16 | 0.95, 1.41 | 1.37 | 0.90, 2.09 | 1.37 | 0.90, 2.09 | 1.34 | 0.88, 2.04 |
| 2010-2011 | 1.51 | 1.24, 1.83 | 1.66 | 1.02, 2.69 | 1.66 | 1.02, 2.70 | 1.61 | 0.99, 2.61 |
| 2012-2014 | 1.80 | 1.49, 2.17 | 1.63 | 0.90, 2.98 | 1.65 | 0.90, 3.01 | 1.61 | 0.88, 2.93 |
| **Year of birth** |  |  |  |  |  |  |  |  |
| Before 2000 | 1 |  | 1 |  | 1 |  | 1 |  |
| 2000-2003 | 1.07 | 0.94, 1.21 | 1.39 | 0.98, 1.98 | 1.38 | 0.97, 1.96 | 1.37 | 0.96, 1.95 |
| 2004-2005 | 0.94 | 0.78, 1.12 | 1.49 | 0.86, 2.59 | 1.53 | 0.88, 2.65 | 1.50 | 0.86, 2.61 |
| 2006-2007 | 1.28 | 1.04, 1.56 | 2.00 | 1.01, 3.97 | 2.05 | 1.03, 4.05 | 2.06 | 1.04, 4.08 |
| 2008 and beyond | 1.29 | 1.01, 1.65 | 1.93 | 0.83, 4.50 | 1.97 | 0.85, 4.58 | 2.01 | 0.87, 4.68 |
| **Age at ART start in years** | 1.06 | 1.04, 1.08 | 1.06 | 0.99, 1.14 | 1.07 | 0.99 1.14 | 1.07 | 1.00, 1.15 |

^$^Children born before 2000 with unknown PMTCT exposure (n=782) assumed to get no PMTCT as no PMTCT available in the public sector before 2000 *Odds Ratios. ** Confidence Intervals

**S6 Table: Univariable models of association between PMTCT exposure and VL >400 copies/ml (viral non-suppression) between 6-18 months on ART.**

|  | **Univariable associations** | | **No assumptions made for unknown PMTCT^$^** | | **Assumption I: Unknown got PMTCT according to yob** | | **Assumption II: Unknown assumed not to have got any PMTCT** | |
| --- | --- | --- | --- | --- | --- | --- | --- | --- |
| **Patient characteristics at ART start** | **Crude OR^*^** | **95% CI^**^** | **Adjusted OR^*^** | **95% CI^**^** | **Adjusted OR^*^** | **95% CI^**^** | **Adjusted OR^*^** | **95% CI^**^** |
| **PMTCT exposure status** |  |  |  |  |  |  |  |  |
| No | 1 |  | 1 |  | 1 |  | 1 |  |
| Yes | 0.95 | 0.78, 1.17 | 1.04 | 0.82, 1.33 | 0.83 | 0.70, 0.99 | 1.12 | 0.88, 1.42 |
| Unknown | 0.74 | 0.66, 0.84 | 0.79 | 0.66, 0.93 |  |  |  |  |
| **Sex** |  |  |  |  |  |  |  |  |
| Male | 1 |  | 1 |  | 1 |  | 1 |  |
| Female | 0.87 | 0.78, 0.96 | 0.86 | 0.77, 0.97 | 0.86 | 0.77, 0.97 | 0.87 | 0.77, 0.98 |
| **WHO Stage** |  |  |  |  |  |  |  |  |
| Stages 1&2 | 1 |  |  |  |  |  |  |  |
| Stages 3&4 | 1.02 | 0.90, 1.17 |  |  |  |  |  |  |
| **WHO-defined Immunosuppression** |  |  |  |  |  |  |  |  |
| No | 1 |  | 1 |  | 1 |  | 1 |  |
| Yes | 2.68 | 2.33, 3.09 | 2.78 | 2.41, 3.22 | 2.78 | 2.40, 3.21 | 2.78 | 2.40, 3.21 |
| **Calendar Year of ART start** |  |  |  |  |  |  |  |  |
| 2004-2005 | 1 |  | 1 |  | 1 |  | 1 |  |
| 2006-2007 | 0.82 | 0.68, 1.00 | 0.79 | 0.62, 0.99 | 0.79 | 0.63, 0.99 | 0.77 | 0.61, 0.96 |
| 2008-2009 | 1.09 | 0.91, 1.30 | 1.06 | 0.83, 1.45 | 1.08 | 0.84, 1.38 | 1.03 | 0.81, 1.32 |
| 2010-2011 | **1.53** | **1.28, 1.83** | **1.49** | **1.11, 2.01** | 1.51 | 1.12, 2.03 | 1.44 | 1.07, 1.94 |
| 2012-2014 | 1.59 | 1.33, 1.89 | 1.32 | 0.91, 1.91 | 1.33 | 0.92, 1.94 | 1.29 | 0.89, 1.87 |
| **Year of birth** |  |  |  |  |  |  |  |  |
| Before 2000 | 1 |  | 1 |  | 1 |  | 1 |  |
| 2000-2003 | 1.04 | 0.92, 1.17 | 1.11 | 0.89, 1.40 | 1.11 | 0.88, 1.39 | 1.09 | 0.87, 1.36 |
| 2004-2005 | 0.97 | 0.82, 1.14 | 1.01 | 0.71, 1.45 | 1.04 | 0.73, 1.50 | 1.01 | 0.70, 1.45 |
| 2006-2007 | 1.29 | 1.07, 1.56 | 1.30 | 0.83, 2.03 | 1.33 | 0.85, 2.08 | 1.33 | 0.85, 2.07 |
| 2008 and beyond | 1.29 | 1.02, 1.62 | 1.41 | 0.81, 2.47 | 1.46 | 0.83, 2.54 | 1.47 | 0.84 2.57 |
| **Age at ART start in years** | 1.05 | 1.03, 1.07 | 1.03 | 0.98 1.08 | 1.03 | 0.98, 1.07 | 1.04 | 0.99, 1.08 |

^$^Children born before 2000 with unknown PMTCT exposure (n=782) assumed to get no PMTCT as no PMTCT available in the public sector before 2000 *Odds Ratios. ** Confidence Intervals

**S7 Fig 1: Number of children by PMTCT exposure status in the different assumptions made**
